# Synergizing Against RAS: The Evolving Landscape of Targeted Combinations in Pancreatic Cancer

**DOI:** 10.3390/cancers18213519

**Published:** 2026-11-02

**Authors:** Aditya K. Ghosh, Khalid A. Jazieh, Hao Xie, Marie A. Parish, Hani M. Babiker, Tanios S. Bekaii-Saab, Ajit H. Goenka, Keith L. Knutson, Akhilesh Pandey, Martin E. Fernandez-Zapico, Ryan M. Carr, Ben George

**Affiliations:** 1 Division of General Internal Medicine, Mayo Clinic, Rochester, MN 55905, USA; ghosh.aditya@mayo.edu; 2 Department of Oncology, Mayo Clinic, Rochester, MN 55905, USAparish.marie@mayo.edu (M.A.P.);; 3 Department of Oncology, Mayo Clinic, Jacksonville, FL 32224, USA; 4 Department of Oncology, Mayo Clinic, Scottsdale, AZ 85259, USA; 5 Department of Radiology, Mayo Clinic, Rochester, MN 55905, USA; 6 Department of Immunology, Mayo Clinic, Jacksonville, FL 32224, USA; 7 Department of Laboratory Medicine and Pathology, Mayo Clinic, Rochester, MN 55905, USA

**Keywords:** pancreatic ductal adenocarcinoma, RAS inhibitors, precision oncology, targeted therapy, clinical trials

## Abstract

Pancreatic ductal adenocarcinoma (PDAC), the most common histological subtype of pancreatic cancer, remains one of the most lethal solid malignancies, with most patients presenting with advanced disease and limited therapeutic options. Activating *KRAS* mutations are present in over 90% of PDACs and drive tumor initiation, progression, metabolic reprogramming, and immune evasion, making *KRAS* a central therapeutic target. Advances in drug development have transformed RAS from a historically “undruggable” oncogene into a clinically actionable target through the development of mutation-selective inhibitors, pan-RAS inhibitors, targeted degraders, and RNA-based therapies. The 2026 approval of daraxonrasib by the FDA for metastatic pancreatic cancer marked the first regulatory approval of a RAS-targeted inhibitor in pancreatic ductal adenocarcinoma and validated RAS blockade as a clinically actionable therapeutic strategy. This review highlights emerging combination strategies designed to maximize the therapeutic potential of RAS inhibition in PDAC. Early clinical studies combining *KRAS*-targeted therapies with standard chemotherapy have demonstrated encouraging response rates and have led to multiple ongoing phase III trials. As the field moves toward precision oncology, biomarker-driven patient selection, circulating tumor DNA monitoring, and adaptive clinical trial designs will be essential for translating these advances into meaningful survival benefits. Collectively, rational combination strategies represent the next frontier in *KRAS*-directed therapy and hold the greatest promise for improving outcomes in PDAC.

## Simple Summary

Pancreatic cancer is one of the deadliest cancers with limited treatment options. More than 90% of pancreatic cancers harbor changes in *KRAS*, a major driver of tumor growth. Recent advances have transformed *KRAS* from a target once considered “undruggable” into one that can be targeted through several novel mechanisms. However, *KRAS* inhibition alone will have limited long-term success because tumors develop ways to bypass treatment and continue growing. This review summarizes emerging synergistic strategies designed to improve the effectiveness of *KRAS*-targeted therapies. These combination strategies represent an important next step toward achieving deeper, more durable responses and improving outcomes for patients with pancreatic cancer.

## 1. Introduction

### 1.1. Pancreatic Cancer: Epidemiology and Burden of Disease

Pancreatic ductal adenocarcinoma (PDAC) has rising global incidence driven in part by modifiable risk factors such as smoking, obesity, diabetes, and metabolic disease [1]. PDAC is projected to become the second leading cause of cancer-related mortality by 2035, with approximately 67,530 new cases expected in the United States in 2026 [2,3,4]. Owing to the absence of effective screening strategies and the typically asymptomatic nature of early disease, most patients present with locally advanced or metastatic cancer, and at most 20% are eligible for potentially curative surgical resection at diagnosis [5]. Cytotoxic chemotherapy remains the mainstay of treatment for most patients; however, substantial toxicity and modest long-term benefit remain a challenge [5]. The dense desmoplastic stroma and profoundly immunosuppressive tumor microenvironment that characterize PDAC contribute to poor drug delivery and limited therapeutic efficacy [5]. Although overall 5-year survival has increased to approximately 13%, these gains have been driven almost entirely by improvements in localized disease, where survival has risen from 24% to 46%. In contrast, outcomes for metastatic disease have improved only marginally from 2% to a little over 3% over the same period [6]. Together, these sobering statistics underscore the persistent limitations of current treatment paradigms and the urgent need for more effective, biomarker-driven therapeutic strategies.

### 1.2. The KRAS Paradigm

Activating mutations in *KRAS* drive over 90% of pancreatic ductal adenocarcinomas (PDACs), functioning as the central mediator of tumor initiation, progression, metabolic reprogramming, and immune evasion [7,8]. Despite this compelling biologic and therapeutic rationale, *KRAS* has eluded drug development for decades. Its smooth molecular surface, exceptionally high affinity for GTP and GDP, and structural homology to wild-type proteins established it as the prototypical “undruggable” target. Consequently, early therapeutic strategies focused on downstream effectors—such as RAF, MEK, and ERK—but yielded limited success in PDAC due to adaptive pathway reactivation and dose-limiting toxicities [7]. The advent of mutation-selective *KRAS* inhibitors and broader RAS-directed therapies has finally overcome these biochemical barriers, ushering in a new era of targeted treatments.

The discovery of the switch-II pocket marked a critical paradigm shift, enabling the first successful allele-specific inhibition of *KRAS* G12C by trapping the protein in its inactive, GDP-bound state (OFF) [9]. This proof of principle catalyzed a rapid expansion of RAS-directed therapeutics, and the field has since evolved far beyond G12C inhibition to encompass a sophisticated arsenal of therapeutic mechanisms [7]. RAS therapeutics now encompass mutant-selective “OFF-state” inhibitors that stabilize GDP-bound *KRAS*, newer RAS(ON) inhibitors that target the active GTP-bound form of RAS through tri-complex formation with intracellular chaperone proteins, allowing inhibition across a wider spectrum of *KRAS* mutations, and targeted protein degradation via proteolysis targeting chimeras (PROTACs) [7]. Collectively, these mechanistic innovations have transformed the RAS therapeutic landscape, providing myriad options to address the wide spectrum of *KRAS* mutations driving PDAC.

Despite the transformative progress represented by direct *KRAS* inhibition, early clinical experience demonstrates that *KRAS*-targeted monotherapy is unlikely to be sufficient for most patients with PDAC. Although mutation-selective and pan-RAS inhibitors have demonstrated meaningful antitumor activity and manageable safety profiles, efficacy is frequently limited by adaptive pathway reactivation, activation of compensatory survival networks, and the emergence of acquired resistance mechanisms that ultimately restore tumor fitness [10]. Consequently, the central challenge is no longer establishing the druggability of RAS but rather identifying and orchestrating synergistic combinations with complementary agents to achieve profound, durable responses. This paradigm shift was underscored by the 2026 approval of daraxonrasib by the FDA for metastatic pancreatic cancer, marking the first regulatory approval of a RAS-targeted inhibitor in pancreatic ductal adenocarcinoma and validating RAS blockade as a clinically actionable therapeutic strategy [11]. This review examines the biologic basis, preclinical rationale, and emerging clinical evidence supporting rational combination strategies designed to circumvent adaptive resistance and maximize the therapeutic potential of RAS inhibition in PDAC.

## 2. The Complexity of *KRAS* Signaling in PDAC

### 2.1. Allelic Diversity of KRAS

Emerging evidence reveals that RAS-mutated PDAC is far from a biologically uniform entity. Although *KRAS* is mutated in 90% of PDAC cases, distinct mutant alleles exert non-uniform functional consequences that fundamentally dictate both patient prognosis and the tumor microenvironment. This structural and functional complexity is underscored by the clinical activity of allele-specific agents. While direct pharmacologic targeting with G12C inhibitors produces meaningful antitumor responses, its overall clinical utility in PDAC is inherently limited by the rarity of the mutation and the rapid onset of adaptive resistance mechanisms [12,13,14]. Because intrinsic GTP hydrolysis rates (G12D > G12V > G12R) determine how quickly a mutated protein transitions into an inactive state, newer non-covalent pan-*KRAS* inhibitors exhibit varying degrees of potency across these sub-populations [15]. The oncogenic potential of *KRAS* mutations is allele-specific, with different *KRAS* variants exhibiting marked heterogeneity in their ability to promote acinar-to-ductal metaplasia, cellular dedifferentiation, and pancreatic tumorigenesis [15]. These mutation-dependent differences appear to arise early in cancer development and are driven by distinct transcriptional, epigenetic, and signaling problems [16]. Ultimately, treating *KRAS*-driven PDAC requires precision, allele-informed strategies that simultaneously address intrinsic cancer cell signaling pathways and variant-specific TME remodeling.

### 2.2. RAS Inhibition (As Monotherapy) in PDAC

Elucidating the mechanisms of *KRAS*-dependent tumor survival has driven the rapid expansion of RAS-targeted therapies in PDAC. While clinically available options remain limited, single-agent efficacy in RAS inhibition has been demonstrated by the *KRAS* G12C inhibitors, Sotorasib, Adagrasib, Olomarisib, and Divarisib, for the small subset of G12C-mutant PDAC patients, alongside the broad pan-RAS(ON) inhibitor Daraxonrasib [12,13,17,18,19,20]. Given its high prevalence and historical recalcitrance to targeted therapy, *KRAS* G12D remains a focus of current PDAC drug development. Multiple mechanistically distinct G12D-directed strategies are now under clinical evaluation, including a robust wave of mutant-selective G12D inhibitors (such as RMC-9805, INCB 162734, HRS 4642, and GFH375), targeted protein degradation (setidegrasib), and localized RNA silencing (siG12D-LODER) [21,22]. Additional allele-specific inhibitors (RMC-5127, *KRAS* G12V) are also in clinical development. While toxicity remains a concern, emerging pan-*KRAS* inhibitors aim to address the molecular heterogeneity of *KRAS* alterations in PDAC, collectively representing a comprehensive, multi-pronged approach to address *KRAS*-driven disease [23,24].

### 2.3. Downstream Signaling Networks

Mutant *KRAS* drives PDAC survival through intricate downstream signaling cascades that adapt based on the distinct biochemical and variant-specific properties of different *KRAS* alleles. GTP-bound *KRAS* primarily engages the RAF/MEK/ERK cascade, which serves as the dominant driver of PDAC initiation and maintenance by regulating broad transcriptional programs necessary for cellular proliferation; notably, constitutive MEK/ERK signaling is sufficient to restore tumor growth even when *KRAS* is inhibited [25,26]. In parallel, *KRAS* activates the PI3K/AKT/mTOR axis, providing a complementary survival program that drives anabolic metabolism, protein synthesis, and resistance to apoptotic stress [25,27]. These networks do not operate linearly but dynamically cooperate and compensate for one another as an adaptive resistance mechanism [25]. The durability of single-pathway inhibition is severely limited due to this inherent signaling redundancy and plasticity; therefore, sustained therapeutic efficacy against *KRAS*-driven tumors necessitates co-targeting these interconnected downstream effector networks to prevent adaptive resistance.

### 2.4. Mechanisms of Resistance

Despite the transformative potential of single-agent RAS-directed therapies in PDAC, the clinical durability of both allele-specific and pan-RAS inhibitors is fundamentally constrained by both intrinsic (de novo) and acquired (adaptive) resistance mechanisms [28]. De novo resistance is mediated by PDAC’s desmoplastic TME, innate cellular plasticity reflecting an epithelial-to-mesenchymal transition (EMT) state, and baseline reliance on complementary bypass circuits, such as the PI3K/AKT/mTOR and YAP cascades [23,24,28]. Under sustained pharmacologic pressure, adaptive resistance rapidly evolves through integrated genetic and non-genetic escape routes. Genetically, tumors frequently undergo focal *KRAS* copy-number amplifications or acquire secondary *KRAS* mutations (e.g., Y96C, H95D) that either sterically occlude inhibitor binding or biochemically shift the protein toward its active GTP-bound conformation, negating the efficacy of direct covalent and non-covalent inhibitors [22,25]. Non-genetically, adaptive resistance is driven by the relief of MAPK-mediated negative feedback loops, prompting rapid hyperactivation of upstream receptor tyrosine kinases (RTKs)—such as EGFR, ERBB2, and MET—that bypass the inhibited RAS to reactivate downstream effector signaling via parallel RAF isoforms [19,21,28]. Most recently, novel resistance mechanisms to RAS tri-complex inhibitors have been identified wherein tumors can acquire mutations that disrupt formation of the RAS-drug-cyclophilin A complex or strengthen native RAS-RAF signaling [29]. These findings have revealed that cancer cells can evade therapy by destabilizing the molecular glue interaction and suggest next-generation tri-complex inhibitors and combination approaches as potential solutions [29]. Collectively, these mechanisms highlight why single-agent RAS inhibition is functionally insufficient in PDAC, establishing the necessity for rational, multi-targeted combinatorial strategies to achieve durable clinical efficacy.

### 2.5. RAS Inhibition in Combination with Cytotoxic Chemotherapy

Given the clonal heterogeneity and adaptive plasticity of *KRAS*-driven signaling in PDAC, various strategies of combining RAS-targeted therapies with established cytotoxic chemotherapy backbones are being actively investigated (Table 1). Building upon the monotherapy survival benchmark established by the FDA-approved pan-RAS inhibitor Daraxonrasib in the phase III RASolute-302 trial [30], early-phase combination studies with gemcitabine and nab-paclitaxel have demonstrated compelling ORRs (~55%), prompting the first-line phase III RASolute-303 trial [31,32]. Parallel efforts utilizing allele-specific *KRAS* G12D inhibitors are following similar pathways; both the liposomal inhibitor HRS-4642 (63.3% combination ORR) and the selective G12D(ON) inhibitor zoldonrasib (~30% monotherapy ORR) are advancing into phase III combination studies, alongside the ON/OFF inhibitor INCB161734 in the DAWN-303 trial [21,25,32]. Concurrently phase III investigations into mechanistically diverse strategies—such as the *KRAS* G12D targeted protein degrader setidegrasib and the downstream MEK inhibitor atebimetinib (MAPKeeper-301)—are currently underway to optimize therapeutic efficacy, prevent resistance, and extend survival [22,32].

Taken together, the current landscape reflects the transition from validating RAS inhibition as an effective therapeutic strategy to integrating these inhibitors into established treatment paradigms. Ongoing phase III trials will determine whether these combinations can significantly improve survival outcomes without prohibitive cumulative toxicity and will likely serve as a bridge until precision medicine strategies are sufficiently mature to yield robust chemotherapy-free treatment regimens for PDAC.

### 2.6. The Rationale for Vertical Inhibition in PDAC

**Figure 1:**
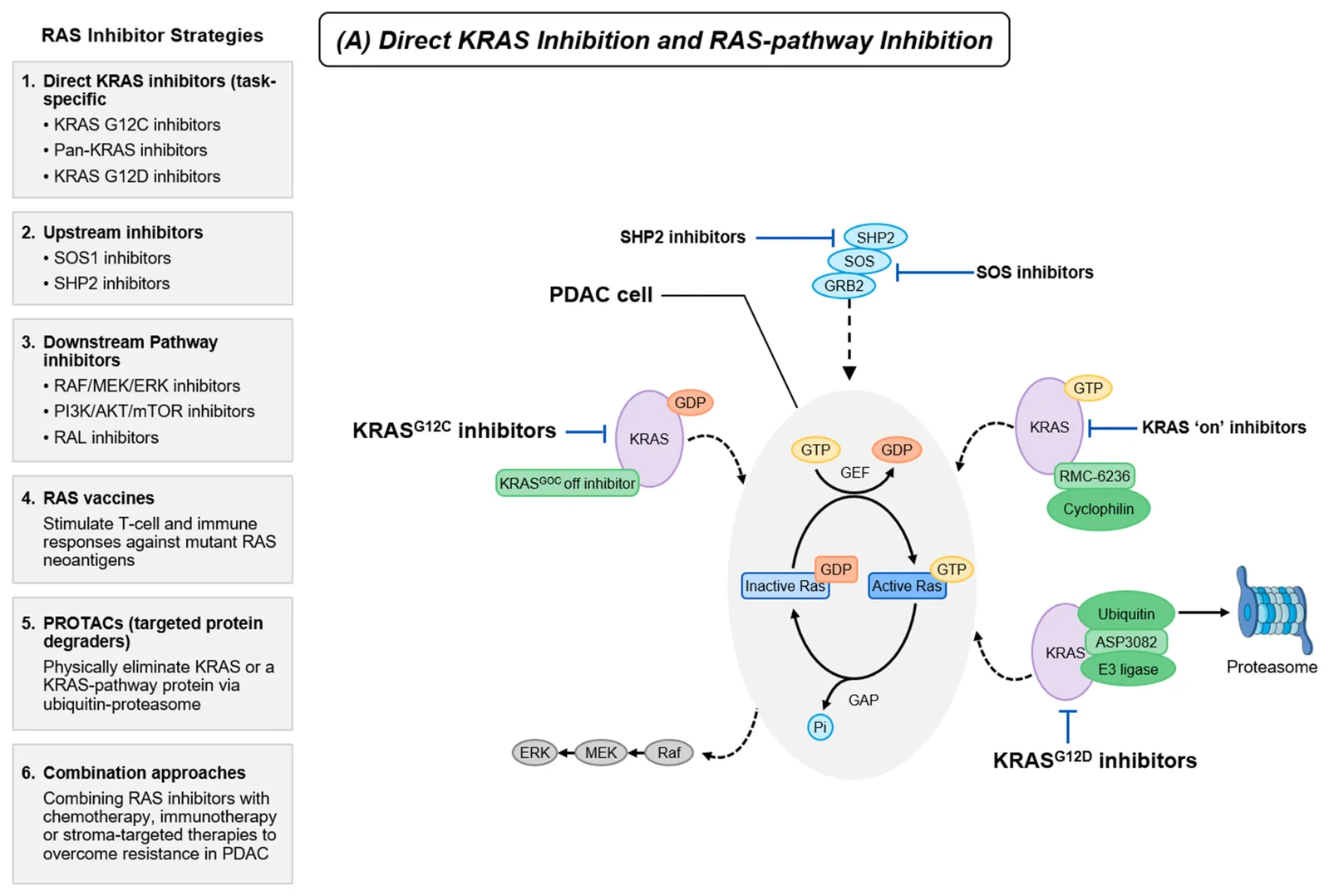

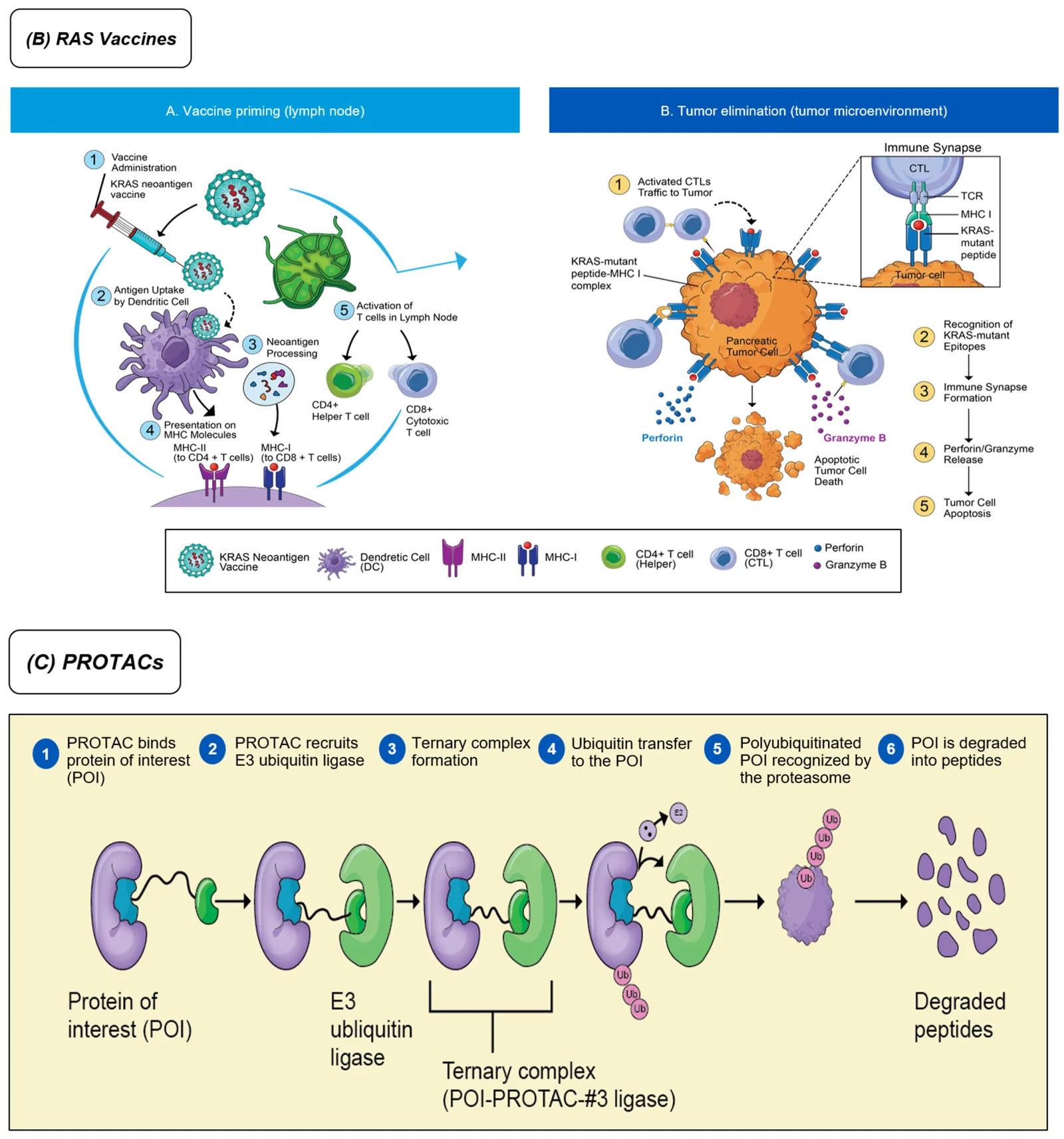
Modalities of RAS Inhibition in PDAC. (**A**) Schematic overview of direct and indirect strategies for targeting *KRAS* signaling in PDAC. *KRAS* cycles between an inactive GDP-bound state and an active GTP-bound state through regulation by guanine nucleotide exchange factors (GEFs), including SOS1, and GTPase-activating proteins (GAPs). RAS therapeutics encompass mutant-selective “OFF-state” inhibitors that stabilize GDP-bound *KRAS*, RAS(ON) inhibitors that target the active GTP-bound form of RAS through tri-complex formation with intracellular chaperone proteins, and targeted protein degradation via proteolysis targeting chimeras (PROTACs Upstream inhibition of SOS1 and SHP2 reduces *KRAS* activation, whereas downstream pathway inhibitors target key signaling nodes including RAF, MEK, ERK, PI3K, AKT, mTOR, and RAL. The left panel summarizes the major therapeutic categories currently being explored for RAS-targeted treatment of PDAC. (**B**) Mechanism of RAS-targeted cancer vaccines. Following vaccine administration, *KRAS*-mutant neoantigens are processed by dendritic cells within lymphoid tissues and presented on major histocompatibility complex (MHC) molecules to CD4^+^ helper and CD8^+^ cytotoxic T cells. Activated T cells expand and traffic to the tumor microenvironment, where recognition of *KRAS*-mutant peptide-MHC complexes on tumor cells leads to immune synapse formation, release of cytotoxic mediators including perforin and granzyme B, and subsequent tumor cell apoptosis. (**C**) Proteolysis-targeting chimeras (PROTACs) as effectors of *KRAS* degradation. PROTACs contain bifunctional ligands that simultaneously bind mutant *KRAS* and E3 ubiquitin ligase, resulting in formation of a ternary complex. This interaction promotes transfer of ubiquitin molecules to the target protein, generating a polyubiquitinated substrate that is recognized and degraded by the proteasome. Degradation of *KRAS* eliminates the oncogenic protein rather than transiently inhibiting its activity, providing a potential strategy to overcome resistance mechanisms associated with conventional small-molecule inhibitors.

*KRAS* blockade reliably induces adaptive resistance through feedback reactivation of the RAS-MAPK signaling cascade; hence, vertical inhibition strategies—targeting both upstream regulators and downstream effectors—have emerged as a mechanistically sound approach to achieve durable tumor suppression (Figure 1).

### 2.7. Targeting Upstream Activators and Downstream Effectors

Suppressing upstream receptor tyrosine kinase (RTK) networks is critical to preventing adaptive RAS-MAPK reactivation, but this feedback is highly context-dependent. For instance, epithelial-differentiated PDAC typically reactivates via EGFR, whereas mesenchymal models rely predominantly on FGFR [33]. Genome-scale screens confirm EGFR as a recurrent resistance node, and combining the RAS(ON) inhibitor RMC-7977 with the EGFR inhibitor erlotinib successfully mitigates rebound ERK activation [34]. However, because single-node RTK targeting is easily circumvented by parallel receptor activation, broader upstream blockade is often required.

SHP2, a critical phosphatase linking RTKs to RAS activation, presents one such target. While the SHP2 inhibitor SDUY104 effectively suppresses *KRAS*-driven PDAC, it simultaneously creates selective pressure for PI3K-AKT bypass signaling, necessitating parallel blockade [35]. Furthermore, early clinical evaluation of vertical SHP2 and ERK blockade (e.g., the phase I SHERPA trial of RMC-4630 and LY3214996) revealed severe, overlapping toxicities—including thrombocytopenia, reduced left ventricular ejection fraction, and acute kidney injury—preventing the achievement of effective target exposures [36]. As an alternative, targeting the guanine nucleotide exchange factor SOS1 offers broader suppression of upstream feedback. By preventing the GDP-to-GTP exchange on RAS across multiple RTK inputs, SOS1 inhibitors act as “multiplier” agents that synergize with direct *KRAS* and MAPK therapies, yielding pan-*KRAS* suppression without being derailed by specific RTK plasticity [37,38].

Complementary to upstream blockades, inhibiting downstream nodes like RAF, MEK, and ERK targets the essential convergence point of oncogenic *KRAS* activity. Preclinical models overwhelmingly support dual-node suppression for superior pathway attenuation [39]. However, the clinical translation of downstream vertical combinations is fundamentally constrained by shared, on-target toxicities. Because MAPK signaling is indispensable for normal epithelial homeostasis, simultaneously inhibiting multiple nodes within the same cascade inevitably narrows the therapeutic window.

**Figure 2:**
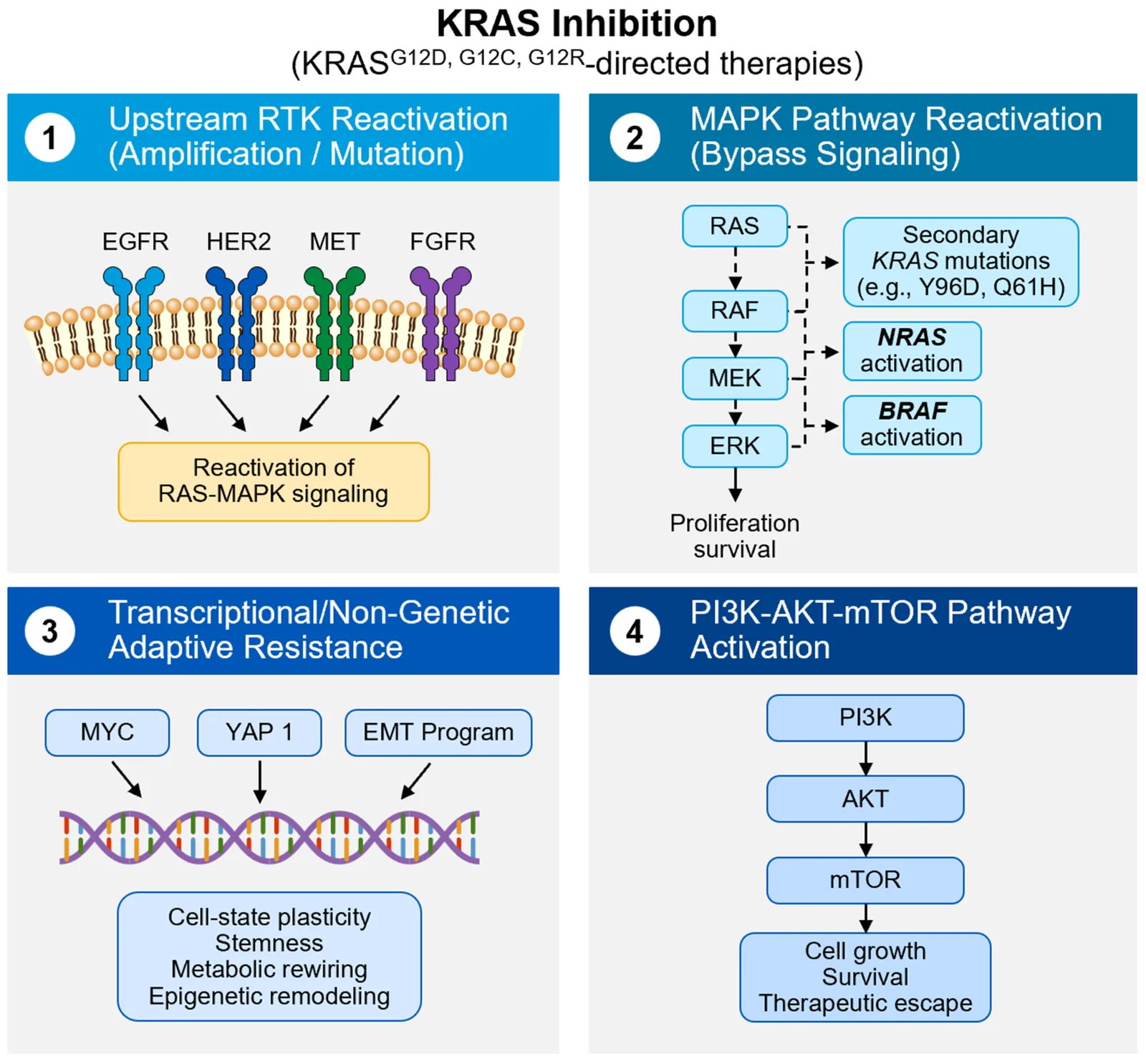
Mechanisms of resistance to *KRAS* inhibition in PDAC. Resistance to *KRAS*-directed therapies in PDAC arises through diverse genetic and non-genetic adaptations that restore oncogenic signaling and promote tumor survival despite pharmacologic inhibition of mutant *KRAS*. (1) Upstream receptor tyrosine kinase (RTK) reactivation. Amplification, overexpression, or activating alterations in upstream RTKs, including epidermal growth factor receptor (EGFR), human epidermal growth factor receptor 2 (HER2/ERBB2), mesenchymal–epithelial transition factor (MET), and fibroblast growth factor receptor (FGFR), can restore RAS-MAPK signaling despite *KRAS* inhibition. (2) MAPK pathway reactivation through bypass signaling. Cancer cells can circumvent *KRAS* inhibition through secondary alterations that restore signaling along the RAF-MEK-ERK cascade. Mechanisms include secondary *KRAS* mutations, activation of alternative RAS isoforms such as NRAS, and activating BRAF alterations, resulting in re-establishment of MAPK signaling, continued tumor proliferation and survival. (3) Transcriptional and non-genetic adaptive resistance. Resistance can emerge through transcriptional reprogramming independent of acquired genomic alterations. Activation of oncogenic transcriptional regulators such as MYC and YAP1, induction of epithelial–mesenchymal transition (EMT) programs, cellular plasticity, stemness phenotypes, metabolic rewiring, and epigenetic remodeling facilitate adaptation to *KRAS* inhibition and promote therapeutic escape. (4) PI3K-AKT-mTOR pathway activation. Compensatory activation of the PI3K-AKT-mTOR signaling axis provides an alternative survival pathway that bypasses *KRAS* dependence.

The clinical reality of profound dermatologic, gastrointestinal, and mucosal toxicities routinely forces dose reductions or treatment interruptions for RAS, MEK, and ERK inhibitors [40,41]. As a result, combining these agents amplifies shared toxicities, and the intensity of pathway inhibition safely achieved in human patients falls substantially short of the threshold required for durable suppression of resistance networks [39]. Ultimately, the success of vertical RAS-MAPK inhibition in PDAC relies not merely on establishing mechanistic synergy in preclinical models, but on innovating drug delivery, sequencing, or mutant-selectivity to meaningfully widen the therapeutic window (Figure 2).

To circumvent the narrow therapeutic window and dose-limiting toxicities inherent to vertical MAPK cascade blockade, targeting parallel networks—most prominently the PI3K-AKT axis—has emerged as a compelling framework to overcome adaptive resistance in PDAC. Far from acting as a passive downstream effector of mutant *KRAS*, PI3K functions as a master regulator of adaptive feedback; Ge and colleagues demonstrated that PI3K promotes resistance through formation of GAB1-mediated wild-type RAS signaling complexes that reactivate MAPK signaling through EGFR-, SHP2-, and SOS1-dependent mechanisms. Importantly, PI3K inhibition restored sensitivity to mutant-selective *KRAS* inhibitors, including in models harboring activating PIK3CA alterations, supporting a mechanistic rationale for combined *KRAS*-PI3K targeting [42].

Additionally, studies suggest that PI3K dependence may be particularly relevant in aggressive basal-like and mesenchymal PDAC subtypes. Köse and colleagues showed that these tumors exhibit heightened PI3K-AKT signaling and undergo substantial PI3K-mediated MAPK rewiring following *KRAS* perturbation. In both patient-derived and in vivo models, combination treatment with the multiselective RAS(ON) inhibitor RMC-6236 and PI3Kα/δ inhibition produced synergistic antitumor effects, while the investigators also identified a PI3Kα/δ-SUMO codependency that may represent an additional therapeutic vulnerability [43]. These findings further support the concept that resistance to *KRAS* inhibition is frequently mediated through signaling networks that extend beyond the canonical MAPK pathway.

Emerging data also indicates that co-targeting PI3K-associated resistance pathways may help overcome acquired resistance to mutation-selective *KRAS* inhibitors. In *KRAS* G12D-resistant PDAC models, it was found that combining the *KRAS* G12D inhibitor MRTX1133 with a dual PI3K/BRD4 inhibitor effectively resensitized resistant tumors. This approach suppressed AKT signaling, reduced YAP1 activation, depleted stem-like tumor cell populations, and favorably remodeled the tumor immune microenvironment, resulting in superior antitumor activity compared with gemcitabine without evidence of significant toxicity in preclinical models [44]. Collectively, these studies suggest that targeting parallel pathways such as PI3K-AKT may provide a more clinically tractable means of overcoming adaptive resistance than escalating inhibition within the MAPK cascade itself, potentially offering a broader therapeutic window while maintaining robust suppression of *KRAS*-driven tumor survival programs.

## 3. Orthogonal Combinations: PRMT5i, Autophagy and Metabolism, Cell Cycle

**Figure 3:**
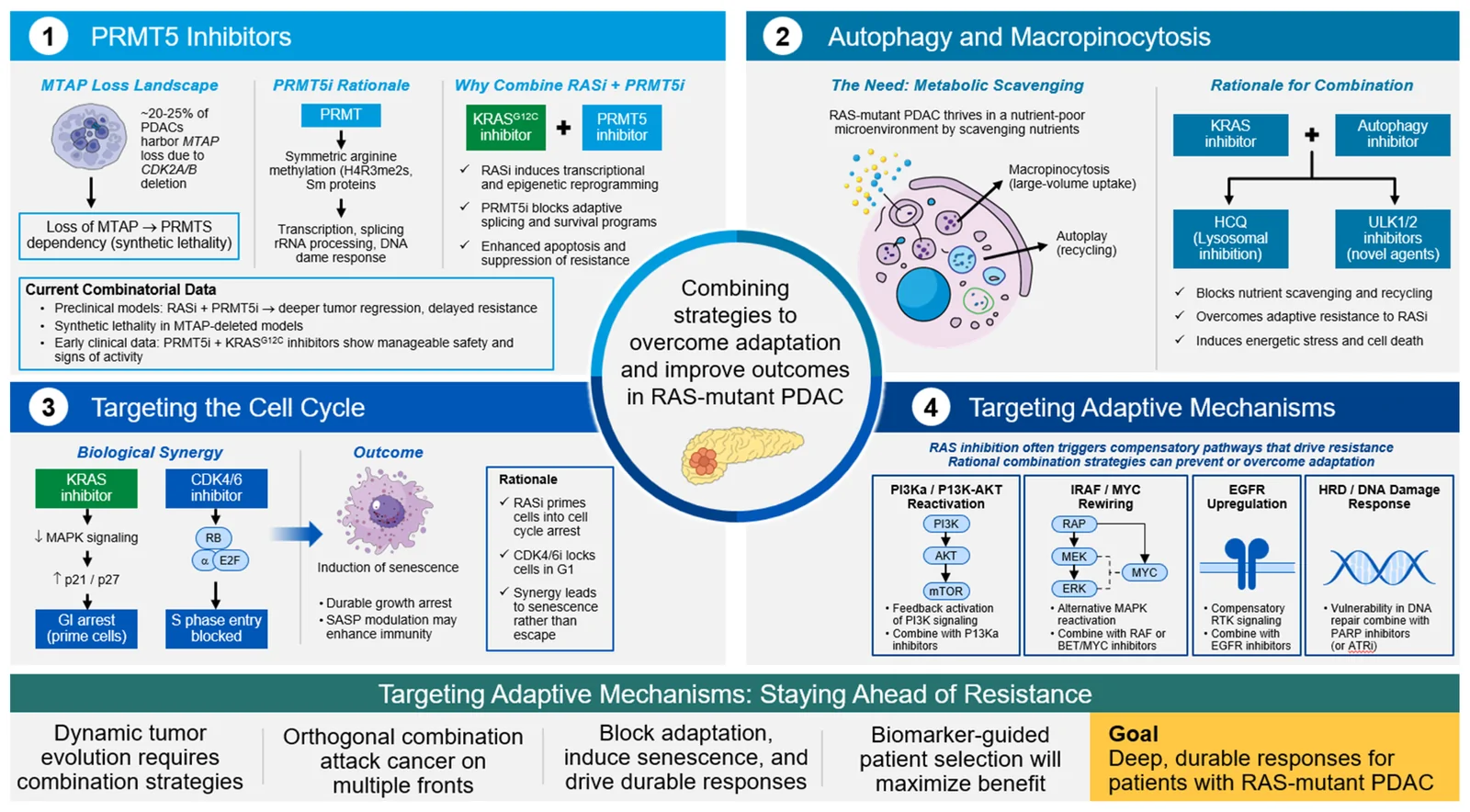
Orthogonal treatment combinations for treatment of RAS-mutant PDAC. (1) *KRAS* inhibitors combined with PRMT5 inhibition. A subset of PDACs harbor homozygous deletion of *MTAP*, creating a synthetic lethal dependency on protein arginine methyltransferase 5 (PRMT5). Combining *KRAS* inhibitors with PRMT5 inhibitors may enhance antitumor activity by suppressing adaptive transcriptional reprogramming and survival pathways induced by *KRAS* blockade. (2) *KRAS* inhibitors combined with autophagy and macropinocytosis inhibition. RAS-mutant PDAC cells rely heavily on autophagy and macropinocytosis to acquire nutrients and maintain metabolic fitness within nutrient-poor tumor microenvironments. *KRAS* inhibition may further increase dependence on these scavenging pathways as an adaptive survival mechanism. Combination strategies utilizing lysosomal inhibitors such as hydroxychloroquine (HCQ) or more selective autophagy pathway inhibitors, including ULK1/2 inhibitors, aim to block nutrient recycling, induce energetic stress, and enhance tumor cell death. (3) *KRAS* inhibitors combined with cell-cycle inhibition. Concurrent targeting of *KRAS* signaling and cell-cycle regulators, particularly cyclin-dependent kinases 4 and 6 (CDK4/6), produces complementary effects on cellular proliferation. *KRAS* inhibition suppresses MAPK signaling and promotes cell-cycle arrest through induction of p21 and p27, whereas CDK4/6 inhibition prevents G1-to-S phase progression through RB-E2F pathway regulation. (4) Combined inhibition of *KRAS* and adaptive resistance pathways. Multiple compensatory signaling pathways can emerge following *KRAS* inhibition, providing opportunities for rational combinatorial approaches. These include co-targeting PI3K-AKT-mTOR signaling to suppress feedback pathway activation; RAF, MEK, ERK, or MYC-directed therapies to prevent MAPK pathway reactivation; EGFR inhibitors to counter receptor tyrosine kinase upregulation; and DNA damage response-targeted agents, including PARP or ATR inhibitors, to exploit vulnerabilities in tumor DNA repair mechanisms.

The limitations of vertical MAPK inhibition necessitate orthogonal combination strategies in pancreatic ductal adenocarcinoma (PDAC). A promising approach exploits the synthetic lethal vulnerability of *MTAP* loss, which co-occurs with *CDKN2A* deletion in 20% to 25% of PDAC tumors. *MTAP* deficiency causes the accumulation of methylthioadenosine (MTA), an endogenous partial inhibitor of protein arginine methyltransferase 5 (PRMT5). This creates a unique therapeutic index by rendering *MTAP*-null tumors heavily dependent on residual PRMT5 activity. This dependency can be targeted via MTA-cooperative PRMT5 inhibitors (e.g., BMS-986504/MRTX1719, AMG 193), MAT2A inhibitors (e.g., IDE397), and emerging PROTAC-based degraders [33,45,46,47] (Figure 3). Combining PRMT5 and *KRAS* inhibition is particularly compelling given the frequent coexistence of *MTAP* loss and *KRAS* mutations in PDAC. Unlike combined MAPK blockade, this orthogonal strategy targets distinct, nonredundant tumor dependencies—*KRAS*-driven oncogenic signaling and PRMT5-mediated epigenetic and RNA regulation—enabling synergistic antitumor activity without overlapping toxicities [45,46]. Preclinical studies robustly validate this combination. A preclinical laboratory study demonstrated that pairing the MTA-cooperative inhibitor BMS-986504 with *KRAS* inhibitors in *MTAP*-deleted, *KRAS*-mutant PDAC models significantly prolonged growth suppression [25]. Mechanistically, *KRAS* and PRMT5 regulate distinct transcriptional programs that converge on shared essential survival genes [25]. Furthermore, another preclinical laboratory study showed that MTA-cooperative PRMT5 inhibition synergizes across multiple MAPK pathway nodes (*KRAS*, RAF, MEK, ERK), generating complete responses and engaging non-cross-resistant mechanisms to combat adaptive resistance [25,48]. While challenges persist regarding intratumoral *MTAP* heterogeneity, biomarker optimization, resistance mechanisms, and systemic toxicities [33,47], combining *KRAS* inhibitors with PRMT5-targeted synthetic lethality remains a highly rational, biologically distinct strategy for PDAC patients harboring concurrent *KRAS* mutations and *MTAP* deletions [46,49].

In *KRAS*-mutant PDAC, the hypovascular and nutrient-deprived tumor microenvironment necessitates a reliance on metabolic scavenging pathways—specifically autophagy and macropinocytosis—for survival [50,51]. Oncogenic *KRAS* drives constitutively elevated autophagic flux and macropinocytosis. Crucially, suppressing *KRAS*-MAPK signaling triggers compensatory nutrient-scavenging programs, creating a strong rationale for combining *KRAS* inhibitors with metabolism-directed therapies to deepen responses and prevent resistance [50,51]. Beyond metabolic support, autophagy facilitates chemoresistance and immune evasion, notably through NBR1-mediated degradation of MHC-I. Because traditional lysosomal inhibitors like hydroxychloroquine exhibit narrow pharmacodynamic effects, next-generation strategies are shifting toward selective ULK1/2 and autophagy receptor inhibition [50,51]. Similarly, macropinocytosis fuels tumor metabolism via extracellular protein catabolism and supports stromal remodeling by maintaining myofibroblastic cancer-associated fibroblasts (myCAFs) under nutrient stress [52]. Therapeutic disruption of this process spans from inhibiting macropinosome formation to inducing methuosis, a cell death pathway driven by dysfunctional macropinocytosis [53]. Combining *KRAS*-MAPK inhibition with the disruption of these lysosomal and autophagic pathways has shown significant translational promise. Preclinically, dual blockade of the RAS-MAPK pathway and PIKfyve, a key endolysosomal trafficking regulator, yields synergistic antitumor activity by impairing adaptive lysosomal responses [54]. Clinically, this orthogonal approach is being evaluated in a phase II trial combining ERK and autophagy inhibition for metastatic PDAC [55].

Targeting cell-cycle regulation is a compelling orthogonal strategy in the treatment of PDAC. Because *KRAS* signaling drives cyclin D-CDK4/6 activity and *CDKN2A* loss is nearly universal, PDAC exhibits unchecked cell-cycle progression. Combining *KRAS* and CDK4/6 inhibitors re-establishes the RB-mediated G1/S checkpoint while suppressing mitogenic signaling, exploiting a shared dependency without the overlapping toxicities of vertical MAPK blockade [56,57]. Crucially, this co-targeting extends beyond simple cytostasis by inducing a robust senescence program. A preclinical laboratory study demonstrated that combined MEK and CDK4/6 inhibition triggers a senescence-associated secretory phenotype (SASP), driving profound tumor remodeling through vascular reorganization, enhanced drug delivery, and favorable immune microenvironment alterations [56]. Corroborating this, a separate preclinical laboratory study showed that concurrent MEK and CDK4/6 inhibition yields durable senescence and prolonged survival in preclinical PDAC models [58]. Consequently, CDK4/6 inhibitors have emerged as highly promising, biologically rational combination partners for next-generation mutation-selective and pan-RAS inhibitors [57]. The therapeutic landscape for *KRAS*-mutant PDAC is shifting away from exclusive reliance on vertical MAPK pathway blockade. Emerging orthogonal strategies increasingly pair *KRAS* inhibitors with agents that exploit independent tumor dependencies, including PRMT5-mediated epigenetic vulnerabilities, metabolic scavenging, and cell-cycle dysregulation. By targeting these complementary mechanisms of adaptation and resistance, combinatorial approaches aim to establish a more durable and clinically tolerable framework for PDAC treatment.

## 4. Synergizing with the Tumor Microenvironment (TME)

**Figure 4:**
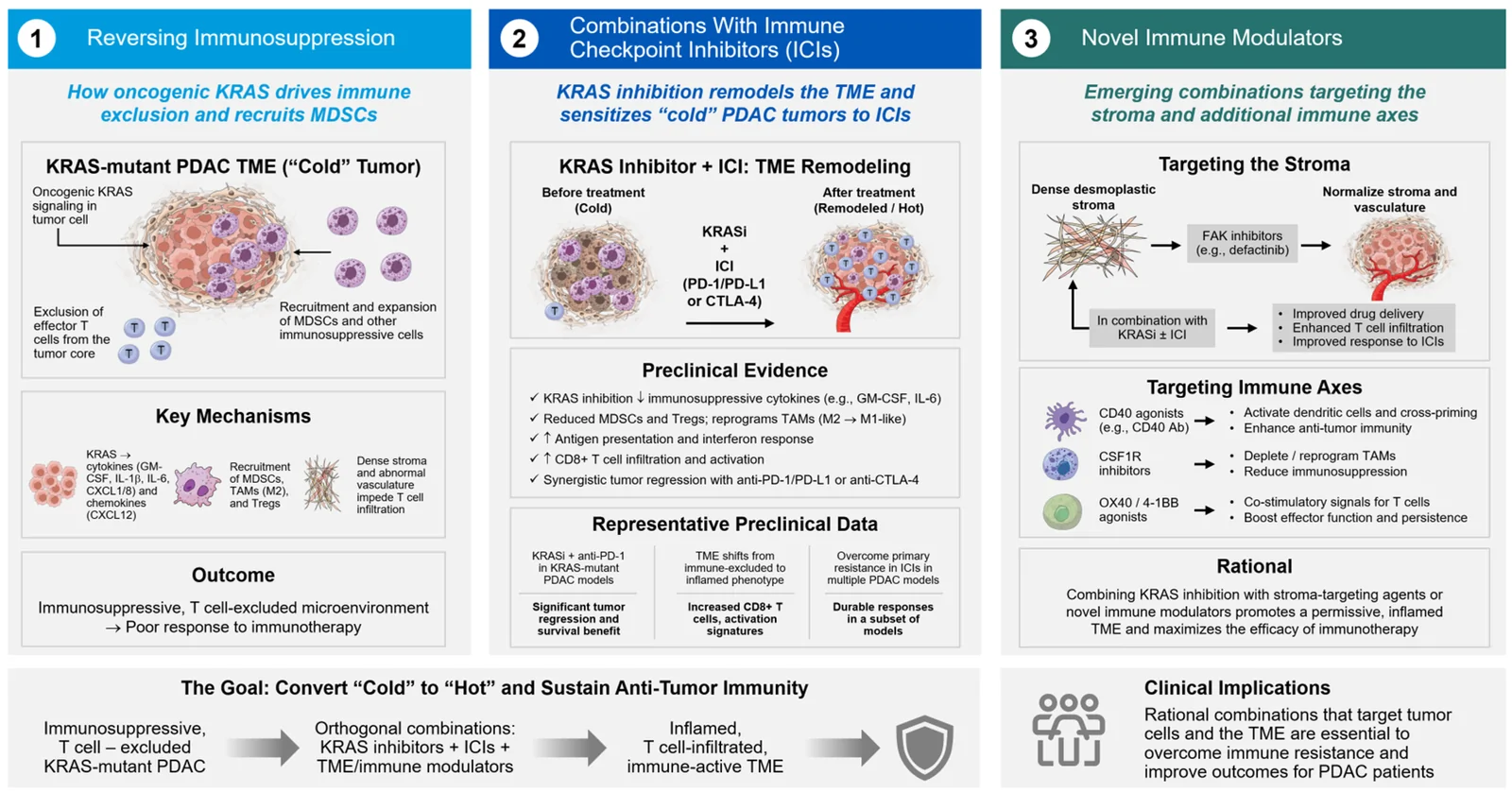
Synergizing with the tumor microenvironment (TME) in PDAC. (1) Reversing *KRAS*-driven immunosuppression. Oncogenic *KRAS* signaling promotes the development of an immunosuppressive tumor microenvironment through secretion of cytokines and chemokines, including GM-CSF, IL-1β, IL-6, CXCL1/8, and CXCL12. These factors recruit myeloid-derived suppressor cells (MDSCs), tumor-associated macrophages (TAMs), and regulatory T cells (Tregs), while the dense desmoplastic stroma and abnormal vasculature impede effector T-cell infiltration. The resulting immune-excluded microenvironment contributes to resistance to immunotherapy and limits endogenous antitumor immune responses. (2) Combination of *KRAS* inhibitors with immune checkpoint inhibitors (ICIs). *KRAS* inhibition can remodel the tumor microenvironment by reducing immunosuppressive cytokine production, decreasing MDSC and Treg populations, repolarizing macrophages toward a pro-inflammatory phenotype, and enhancing antigen presentation and interferon signaling. These changes promote infiltration and activation of CD8+ cytotoxic T cells and may sensitize previously “cold” PDAC tumors to immune checkpoint blockade, including PD-1/PD-L1 and CTLA-4 inhibitors. Preclinical studies have demonstrated synergistic antitumor activity and durable tumor regression with combined *KRAS* inhibition and immune checkpoint blockade. (3) Novel immune modulators and stromal-targeting strategies. Emerging therapeutic approaches aim to further enhance the efficacy of *KRAS* inhibition through modulation of the stromal and immune compartments of the TME. Stromal-targeting therapies, including focal adhesion kinase (FAK) inhibitors, may normalize desmoplastic stroma and tumor vasculature, improving drug delivery and T-cell infiltration. Additional immune-modulating strategies include CD40 agonists to enhance dendritic cell activation and T-cell priming, CSF1R inhibitors to deplete or reprogram immunosuppressive macrophages, and OX40 or 4-1BB agonists to augment T-cell activation, persistence, and effector function.

Oncogenic *KRAS* drives a profoundly immunosuppressive, desmoplastic TME in PDAC. This immune-excluded phenotype is maintained via autophagy-mediated MHC-I degradation, the recruitment of suppressive myeloid populations (e.g., MDSCs, macrophages), and dense stromal barriers that restrict CD8^+^ T-cell infiltration [8,59]. Crucially, these barriers are reversible, allowing targeted *KRAS* inhibition to act as an immunologic primer that sensitizes “cold” PDAC tumors to immune checkpoint blockade (ICB) [59] (Figure 4).

Preclinical studies validate this synergy. In *KRAS* G12D models, the selective inhibitor MRTX1133 reversed immune exclusion by reducing suppressive myeloid cells, reprogramming cancer-associated fibroblasts (CAFs), and restoring intratumoral CD8^+^ T cells—which were required for tumor regression. Combining MRTX1133 with ICB resulted in synergistic tumor eradication [60]. Similarly, single-cell profiling revealed that *KRAS* G12C inhibition promotes mature dendritic cell states and cytotoxic T-cell infiltration, with concurrent anti-PD-1 therapy further expanding effector T-cell clones to improve survival [61].

To fully overcome multifaceted therapeutic resistance, combinatorial therapeutic strategies may benefit from incorporating stromal modulators and immune agonists. Focal adhesion kinase (FAK) inhibition disrupts reciprocal tumor-stroma signaling, reprograms CAFs, and enhances T-cell priming, supporting the clinical evaluation of the FAK inhibitor defactinib alongside RAF-MEK blockade (avutometinib) and chemotherapy [62]. Additionally, combining RAS inhibitors and ICB with immune agonists like CD40 actively drives potent, T-cell-dependent antitumor immunity, particularly in tumors with baseline immune infiltration [63]. Ultimately, multidimensional strategies that simultaneously inhibit oncogenic signaling, remodel the stroma, and activate immune responses represent a highly rational therapeutic frontier for PDAC.

## 5. Clinical Translation and Trial Design Perspectives

Rapid advances in drug discovery have ushered in an era of unprecedented therapeutic opportunities to tackle PDAC; however, their successful clinical translation necessitates a paradigm shift in how patients are selected and clinical trials are structured. Integrating comprehensive tissue-based genomic profiling (CGP) and longitudinal molecular monitoring via circulating tumor DNA (ctDNA) into standard clinical workflows is critical for patient selection, stratification, and deployment of adaptive therapeutic strategies. Routine utilization of minimal residual disease (MRD) assays post-resection and liquid biopsies allows for the real-time, non-invasive interrogation of tumor evolution. This continuous monitoring facilitates the early detection of dynamic, adaptive resistance mechanisms often long before radiographic progression is apparent. This high-resolution temporal data is paramount for rationally sequencing therapies and intercepting resistance early.

To efficiently evaluate these biologically tailored combinations, the oncology community must move beyond traditional, rigidly structured clinical trials and embrace innovative, adaptive platform trials, such as umbrella and basket designs. These agile frameworks allow for the concurrent evaluation of multiple targeted therapies across genetically defined patient cohorts. By utilizing Bayesian statistical models and adaptive randomization, these platforms can rapidly graduate highly active regimens to broader evaluation or swiftly eliminate futile arms, minimizing patient exposure to ineffective and potentially toxic therapies [64,65,66]. Implementing these adaptive platforms in the metastatic setting accelerates the identification of active regimens, while deploying them in the neoadjuvant setting offers the dual benefit of maximizing curative-intent resections and acquiring invaluable on-treatment tissue for reverse-translational mechanistic studies. Ultimately, synergizing high-fidelity biomarker strategies with agile trial designs is crucial for translating preclinical combinatorial promises into durable survival benefits for patients with PDAC.

## 6. Conclusions

Our current state of knowledge suggests that effective targeted therapy for PDAC relies on transitioning from single-agent approaches to rational, multi-nodal pathway suppression. As these sophisticated combinatorial strategies advance through clinical development, investigators must rigorously balance the pursuit of synergistic efficacy against the risk of dose-limiting, overlapping toxicities. Beyond the metastatic setting, integrating these novel therapies into the neoadjuvant and adjuvant spaces is an absolute necessity. Successfully deploying rational targeted combinations in earlier disease stages to eradicate micrometastatic disease can fundamentally transform the therapeutic landscape and substantially increase cure rates for patients with PDAC.

## Figures and Tables

**Table 1:** Current Clinical Trials Evaluating RAS-pathway Inhibitors in Combination with Chemotherapy Backbone for treatment of Pancreatic Ductal Adenocarcinoma.

Trial/Phase	Agent (Class)	Chemotherapy Backbone	Line of Therapy	ORR (%)	mPFS, HR (95% CI)	mOS, HR (95% CI)	Status
RASolute-302 (NCT06625320), Phase III	Daraxonrasib (RMC-6236, pan-RAS[ON])	Monotherapy	2L RAS-mutated metastatic PDAC	31.6	7.3 mo; HR NR (95% CI NR)	13.2 mo vs. 6.6 mo; HR 0.40 (95% CI NR)	FDA approved
NCT05379985, Phase I/II	Daraxonrasib (pan-RAS[ON])	Gemcitabine/nab-paclitaxel	1L metastatic PDAC	~55	NR	NR	Early efficacy data reported
RASolute-303, Phase III	Daraxonrasib (pan-RAS[ON])	Combination chemotherapy (3-arm study)	1L metastatic PDAC	NR	NR	NR	Ongoing
NCT06520488, Phase Ib/II	HRS-4642 (*KRAS* G12D, IV liposomal)	Nab-paclitaxel/gemcitabine	1L *KRAS* G12D-mutant advanced PDAC	63.3	NR	NR	DCR 93.3%; grade ≥ 3 TRAEs 90.3%
Phase III	HRS-4642 (*KRAS* G12D)	Combination chemotherapy	1L *KRAS* G12D-mutated PDAC	NR	NR	NR	Undergoing Phase III evaluation
RASolute-305, Phase III	Zoldonrasib (RMC-9805, *KRAS* G12D[ON])	Combination chemotherapy	1L *KRAS* G12D-mutated PDAC	~30*	NR	NR	Ongoing; chemo + inhibitor vs. chemotherapy alone
DAWN-303, Phase III	INCB161734 (*KRAS* G12D ON/OFF inhibitor)	Combination chemotherapy	1L *KRAS* G12D-mutated PDAC	NR	NR	NR	Ongoing
MAPKeeper-301, Phase III	Atebimetinib (MEK inhibitor)	Combination chemotherapy	1L PDAC	NR	NR	NR	Ongoing
Phase III	Setidegrasib (ASP3082, *KRAS* G12D degrader)	Combination chemotherapy	*KRAS* G12D-mutated PDAC	NR	NR	NR	Phase III underway
KRYSTAL-1/CodeBreaK100	Adagrasib/Sotorasib (*KRAS* G12C)	Monotherapy	Previously treated *KRAS* G12C-mutated PDAC	33 (adagrasib); 21 (sotorasib)	NR	8.0 mo (adagrasib); 6.9 mo (sotorasib)	Included in NCCN guidance

## Data Availability

No new data were created or analyzed in this study. Data sharing is not applicable to this article.
